# Editorial: Insights in brain imaging methods: 2023

**DOI:** 10.3389/fnins.2024.1488845

**Published:** 2024-09-23

**Authors:** Federico Giove, Xi-Nian Zuo, Vince D. Calhoun

**Affiliations:** ^1^MARBILab, Museo storico della fisica e Centro studi e ricerche Enrico Fermi, Rome, Italy; ^2^Neuroimaging Laboratory, Fondazione Santa Lucia IRCCS, Rome, Italy; ^3^State Key Laboratory of Cognitive Neuroscience and Learning, Beijing Normal University, Beijing, China; ^4^Tri-institutional Center for Translational Research in Neuroimaging and Data Science, Georgia State University, Georgia Tech, Emory, Atlanta, GA, United States

**Keywords:** brain, imaging, neuroscience, MRI, light sheet microscopy, MEG, BCI, EEG

Brain imaging has revolutionized our understanding of the human brain, enabling the exploration of its structure and function in great detail. This history of enduring success has its roots in the continuous development of methods that merge new technologies and processing approaches. Indeed, funding bodies like the European Commission, the National Institutes of Health, and the Wellcome Trust have repeatedly included advanced neuroimaging and next-generation imaging modalities among their funding priorities to shape the future of neuroscience.

Methodological advances in brain imaging are thus at the forefront of neuroscience progress. New methods in neuroimaging are crucial not only for neuroscience, but also for improving our understanding of how brain structure and function change in response to disease, injury, or therapeutic intervention. Brain imaging is pivotal in identifying biomarkers of neurodegeneration, enabling earlier and more accurate diagnosis and paving the way for more targeted and effective treatments. By providing insight into an individual's brain structure and function, imaging can help tailor treatments to the needs of each patient. Personalized medicine aims to optimize therapeutic outcomes by taking into account individual variability, and brain imaging is an indispensable tool in achieving this goal.

A reliable biomarker of myelin integrity is critical for further developments in the understanding, diagnosis, and treatment of multiple neurological diseases. By combining MRI and histology in mice, Searleman et al. showed that Ultrashort Echo Time (UTE) MRI is sensitive to myelin loss due to its ability to detect very fast relaxation signals.

Structural and functional connectomics based on magnetic resonance imaging (MRI) is a field in constant expansion, where innovations in signal acquisition, processing, and theoretical modeling are equally important. Li et al. focused on the representation of the microstructural connectome. They argued that the conventional reliance on an adjacency matrix hampers statistical and computational efficiency by inflating dimensionality beyond what is needed and showed that a topologically and biologically informed tree representation preserves information and interpretability while drastically reducing dimensionality.

Functional connectivity (FC) was the focus of the study by Hu et al., who investigated its spatiotemporal modulation during naturalistic stimuli. They found that a naturalistic stimulus (watching a movie) modulates the magnitude but does not change the pattern of connectivity compared to resting conditions. Temporal coherence of fluctuations between subjects, as assessed by inter-subject functional connectivity, was weak during the naturalistic stimuli, indicating that brain fluctuations of different subjects are not synchronous under the same naturalistic condition. Overall, the authors reported improved stability of FC metrics under naturalistic stimuli compared to rest, suggesting that the presentation of naturalistic stimuli may be preferable for performing FC studies in neurological and psychiatric patients.

While MRI-based FC has generated substantial new knowledge at the scale of whole-brain connectivity, and single neurons can be effectively studied by electrophysiological methods, it is still a challenge to close the gap at the mesoscale, the scale of neural circuits. Caznok Silveira et al. review the potential, challenges, and limitations of neuroimaging to investigate connectivity at the mesoscale.

Local assessment of cell number and density is a useful tool for the study of CNS diseases in animal models; however, it is prone to technical biases associated with tissue deformation, selection of sampling sites, and mere errors. Tian et al. reported a multimodal approach based on MR histology and light sheet microscopy, to address this problem, and show that their workflow allows accurate regional counting in a mouse model of aging.

At the other extreme of the spatial scale, brain volume and cortical thickness can be assessed by computational techniques leveraging structural MRI images. Del Giovane et al. briefly examined the effectiveness of current approaches for extracting these metrics from brains with abnormal anatomy, such as those seen in idiopathic Normal Pressure Hydrocephalus. They conclude that the task still requires a degree of manual editing that is necessarily associated with inter-rater variability.

While neuroscientists are normally concerned with populations, individual variations can convey information and are certainly crucial for personalizing treatments. Kampel et al. showed that multivariate time-series classification of MEG time-series performed with random convolutional kernel transformation (ROCKET) allows neuronal fingerprinting, i.e., the identification of single subjects with great accuracy on time-series windows as short as 1 s. This performance is promising for personalized medicine and the development of brain-computer interfaces (BCI).

BCI decoding algorithms can be improved by optimizing the extraction of features from EEG signals. Ma et al. introduced a method for extracting network features from EEG traces, based on directed transfer function and graph theory. The authors showed that their method improves performance in the classification and decoding of motor imagery tasks, thus potentially contributing to increased accuracy and reliability of BCI.

Finally, the transition from research to clinical settings requires standardized procedures. Wang et al. described the optimization of a scoring system based on semiquantitative MRI imaging, common in hospitals, designed to assess Wilson's Disease; they showed improved predictive performance over previously available approaches. In another clinically oriented work, Gao et al. investigated the risk of postoperative cerebral hypoperfusion after revascularization surgery for moyamoya disease using pulsed arterial spin labeling combined with time-of-flight angiography. The researchers showed that the risk can be stratified using non-invasive and safe MRI procedures, without the administration of contrast agents.

While certainly not exhaustive, this Research Topic offers an overview of some of the frontier themes in brain imaging methods. Taken together, the studies clearly suggest that multimodal integration, new acquisition and processing techniques, and validation for clinical applications are interplaying features of brain imaging method development.

